# Patient experiences and perspectives of health service access for carpal tunnel syndrome in Aotearoa New Zealand: a normalisation process theory-informed qualitative study

**DOI:** 10.1186/s12913-024-10871-x

**Published:** 2024-04-13

**Authors:** Miranda Bűhler, Carol Atmore, Meredith Perry, Sue Crengle, Pauline Norris, G. David Baxter

**Affiliations:** 1 Department of General Practice & Rural Health, Dunedin School of Medicine, University of Otago, Dunedin, New Zealand; 2 Centre for Health, Activity and Rehabilitation Research, School of Physiotherapy, University of Otago, PO Box 56, Dunedin, 9010 New Zealand; 3 Centre for Health, Activity and Rehabilitation Research, School of Physiotherapy, University of Otago, Wellington, New Zealand; 4 Ngāi Tahu Māori Health Research Unit, University of Otago, Dunedin, New Zealand; 5 Department of Preventive and Social Medicine, Dunedin School of Medicine, University of Otago, Dunedin, New Zealand; 6 School of Pharmacy, University of Otago, Dunedin, New Zealand

**Keywords:** Health services accessibility, Carpal tunnel syndrome, Qualitative research, Health Equity, Normalisation process theory, Co-design

## Abstract

**Background:**

Early access to care for carpal tunnel syndrome (CTS) can avoid higher rates of surgery and permanent harm yet is often delayed, particularly for populations more likely to underutilise care.

**Objective:**

We sought to explore patient experiences and perspectives of health service access for CTS to inform an equity-focussed co-design of a health service for improving early care access.

**Methods:**

In this Normalisation Process Theory (NPT)-informed qualitative study we conducted semistructured in-depth interviews with 19 adults with experience of CTS. Recruitment prioritised New Zealand Māori, Pasifika, low-income, and rural populations. Data were analysed using deductive then inductive thematic analysis.

**Results:**

We identified five major themes: (1) the ‘Significant Impact of CTS’ of the sense-making and relational work to understand the condition, deciding when to get care, compelling clinicians to provide care, and garnering help from others; (2) ‘Waiting and Paying for Care’– the enacting, relational, and appraising work to avoid long wait times unless paying privately, particularly where quality of care was low, employment relations poor, or injury compensation processes faltered; (3) circumstances of ‘Occupation and CTS Onset’ whereby the burden of proof to relate onset of CT symptoms to occupation created excessive relational and enacting work; (4) the ‘Information Scarcity’ of good information about CTS and the high relational and appraising work associated with using online resources; (5) ‘Negotiating Telehealth Perspectives’ where telehealth was valued if it meant earlier access for all despite the challenges it held for many.

**Conclusion:**

Quality, culturally and linguistically responsive information and communication from clinicians and health services will improve equitable early access to CTS care including realising the potential of telehealth modes of care. Policy changes that reduce individual burden of proof in injury compensation claims processes, enable time off work to attend health appointments, and increase public funding for surgical resources would improve early access to CTS care particularly for Māori and Pacific populations and those in small and rural workplaces. NPT is valuable for understanding where opportunities lie to reduce inequitable delays to accessing care including the impact of racism, particularly for populations more likely to underutilise care.

**Supplementary Information:**

The online version contains supplementary material available at 10.1186/s12913-024-10871-x.

## Introduction

Carpal tunnel syndrome (CTS) is a common musculoskeletal problem for which there is a good consensus on treatment, including the importance of accessing early care [1, 2]. Affecting around one in ten people at some point over their lifetime [3], CTS involves compression of the median nerve at the wrist, causing sensory loss, pain, and functional impairment including lost work days and sleep disturbance [1–4]. Education, advice, and splinting can be effective where symptom duration is less than 1 year [1, 2]; surgical decompression is recommended where symptoms become severe or do not improve [2, 3].

In the New Zealand healthcare system, access to timely care can be difficult; care for musculoskeletal conditions such as CTS is under-resourced [5], in common with many countries [6, 7]. People with CTS must negotiate accessing care that straddles complex public-private interfaces. Although New Zealand has a universal secondary-level public health system with a smaller private system, general practice (GP) is accessed on a subsidised user-pays basis [8] and most allied health (AH) primary care services have no subsidy. Therefore, both primary and second tier services can be out of reach for those unable to pay [9]. Where CTS can be attributed to occupation, the publicly funded Accident Compensation Corporation (ACC) subsidises AH, specialist, and GP care. However, the ACC acceptance rate for CTS is extremely low– only about 25% of claims submitted are recognised, lower for indigenous Māori (23%) and Pacific peoples (people of Pacific ethnicity) (22%) compared with European (28%) ethnicity (ACC, e-mail communication, February 2021).

In addition to access, inequitable outcomes for CTS are also a problem. Māori and Pacific peoples suffer worse outcomes and lower rates of access across musculoskeletal conditions [10–12]. These populations are more likely to be in manual work [13], a risk factor for CTS [3]. Additionally, people with diabetes are five times more likely to develop CTS [14]– and diabetes is nearly three times more common in Māori [15] and four times more common in Pacific peoples [16] than in the general population. Low socioeconomic status, poor access to transport or telecommunications, service cost, and distance are associated with reduced access to health services overall [12, 17, 18]. In New Zealand, there is an obligation that new and existing models of care help eliminate health inequities, including identifying where inequities exist [19].

HealthPathways is a New Zealand-based tool designed to support better quality care through improved integration across primary and secondary services by making expert clinical knowledge, relevant to the local context, available to primary care clinicians using a digital platform [20, 21]. However, outcomes of HealthPathways have seldom been evaluated at the patient level [22, 23] and their impact on inequity is not well understood [23]. Another tool with potential to improve care access for musculoskeletal conditions including CTS [24] is telehealth. Defined here as synchronous telephone or video consultation, telehealth has become widely available since the Covid pandemic. It is likely to remain part of health delivery [25], although concerns persist about its deployment as a panacea for limited health resources [26] and about risk for exacerbating existing health inequities [27]. In developing a local CTS HealthPathway that could optionally incorporate telehealth tools in the south of New Zealand, we undertook a co-design process [28, 29]. This prioritised those more likely to underuse health services to gain an understanding of inequities in access, experience, and outcomes for CTS from the patient perspective. The overarching aim was to develop health services that would be more likely to help eliminate inequities.

**Fig. 1:**
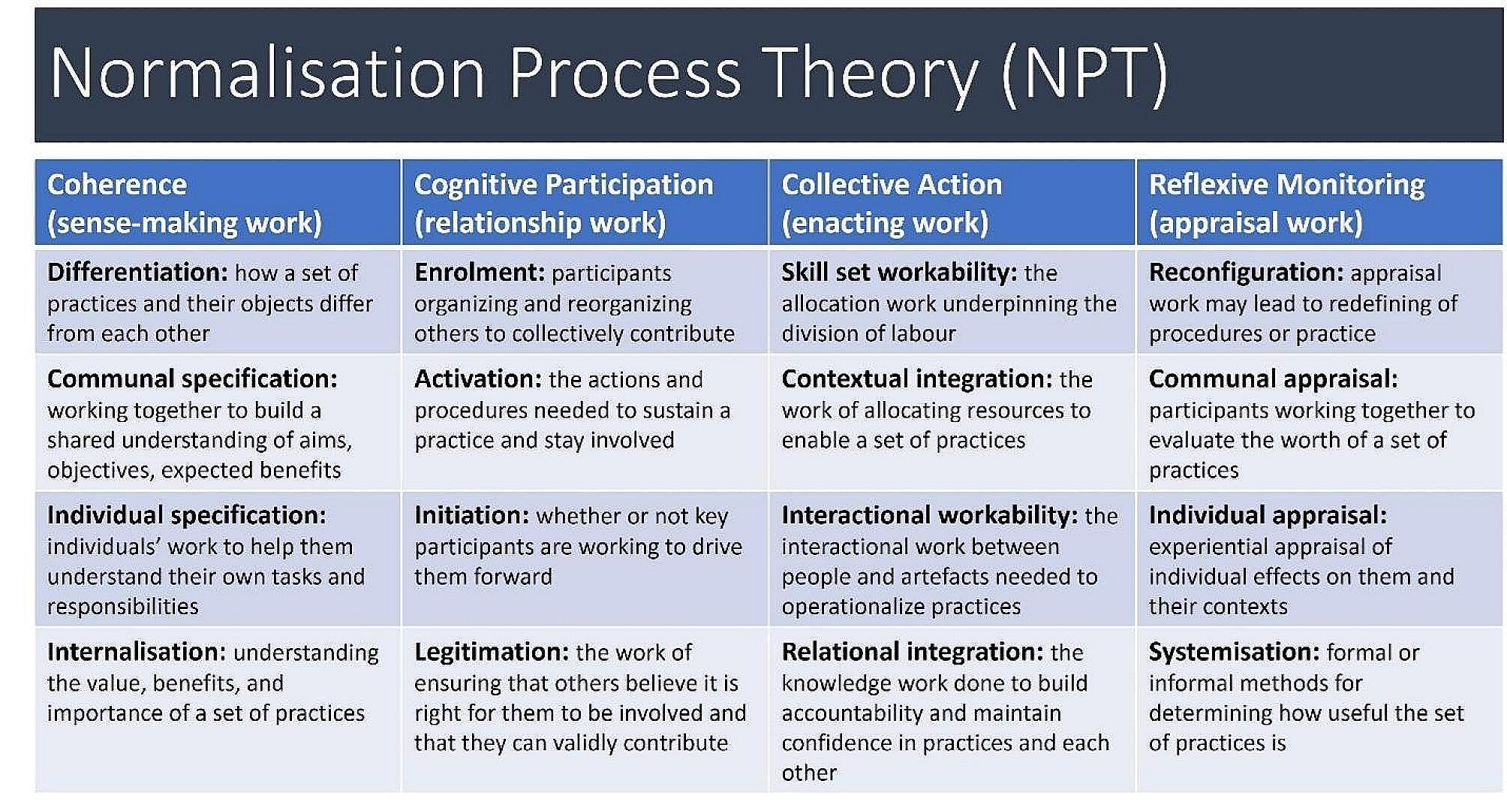
Normalisation process theory domains and constructs [30]

To this end, in the early patient story-gathering phase, we employed an implementation science framework, Normalisation Process Theory (NPT) [30] to help guide the interview schedule and interpretation. A robust mid-range theory, NPT seeks to explain and guide implementation processes, including consideration of how existing or future aspects of a service are likely to impact equity [31, 32], by asking questions about the labour involved, in this case for patients seeking and engaging care, in terms of: (1) sense-making, (2) relationships, (3) taking action, and (4) appraising [30] (Fig. 1). An extension of NPT, the Burden of Treatment Theory [31], was also applied to help understand how patients navigate this work. It examines the interaction between patients’ capacity for action, including the material and cognitive resources at their disposal, and the work that healthcare systems pass on to them.

This study aimed to explore patient experiences and perspectives of health services for CTS using NPT and the Burden of Treatment Theory to inform the care pathway co-design.

The full co-design process and an evaluation of the resulting care pathway will be reported elsewhere.

## Methods

This participatory qualitative study was undertaken within a co-design approach oriented to “working with patients and focussing on experiences” [33]. Ethical approval was obtained from the University of Otago Human Ethics Committee (Health), reference H21/026. Māori consultation was completed with the Ngāi Tahu Research Consultation Committee. All participants provided informed written consent.

**Fig. 2:**
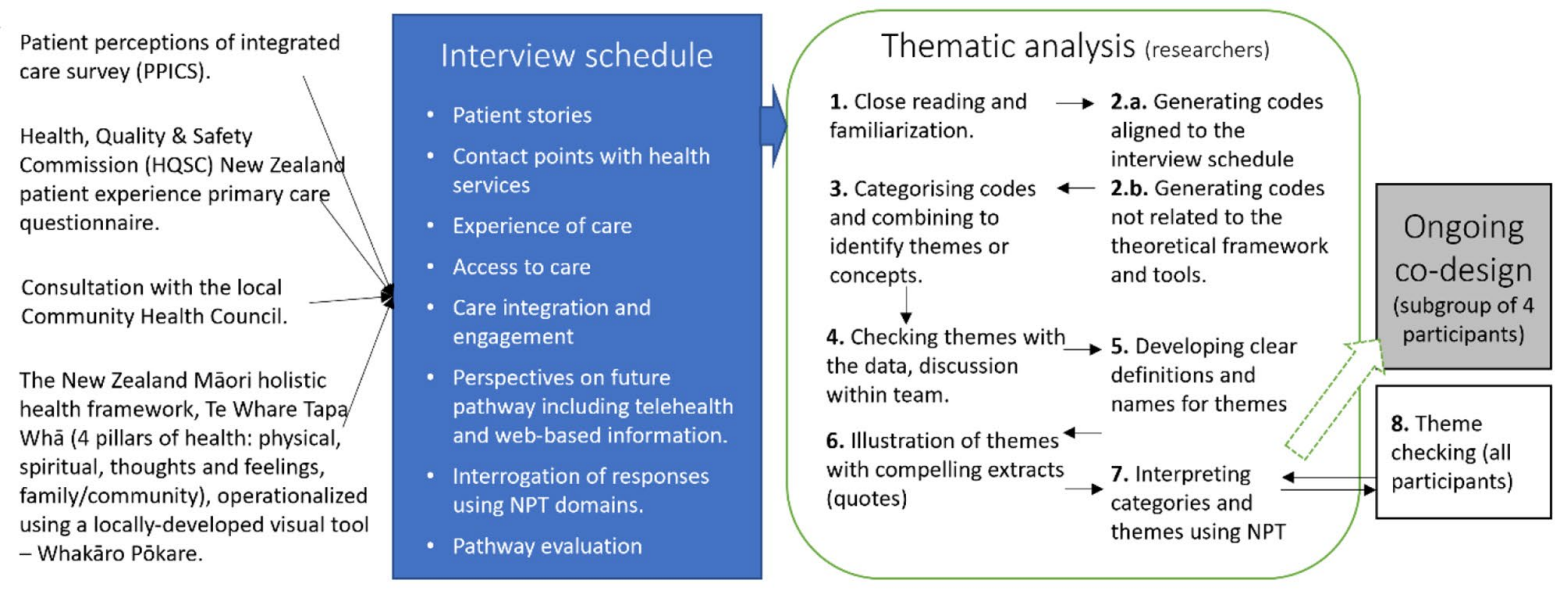
Study methods: interview schedule development, process of thematic analysis, and relationship with co-design process

We conducted semistructured in-depth individual interviews with 19 adults with experience of CTS. The pilot-tested interview schedule covered eight fields of interest (Fig. 2 and Supplemental 1). Interview questions were generated from existing quality of care evaluation instruments [34, 35]. The New Zealand Māori holistic health framework of Te Whare Tapa Whā *[four-pillared house]* [36], represented by a locally developed visual tool [37] was offered to invite participants to reflect on their CTS story and its impact across four pillars of health: physical, spiritual, thoughts and feelings, and family/community.

A minimum of five participants from each of the following key demographic characteristics were sought: Māori ethnicity; Pacific ethnicity; Low-income (using ‘fixed line measure’ [38]), seasonal, zero hours, or out of work; rural domicile [39]. A minimum of five with each of the following care characteristics were sought: CT surgery in the past 12 months; not sought or received care beyond GP consultation with or without medication. Those who received non-pharmacological, non-surgical care completed the total number of participants.

Participants were purposefully recruited from health and non-health settings in the Otago and Southland districts of New Zealand, including GPs, procedure- and wait-lists, Community Health Council, trade unions, marae-based networks, and Pasifika networks, by clinician invitation and paper- or social media-based advertisement. Inclusion and exclusion criteria are outlined in Table 1.

**Table 1:** Inclusion and exclusion criteria

Inclusion	Exclusion
Clinical symptoms^a^ ≥3 months Age ≥ 18 years Resident or working in Otago or Southland. Can communicate in English language independently or with support person. Provide written informed consent	Unclear diagnosis and no nerve conduction study. Cervical radiculopathy Acute trauma past 3 months ≥ 1-year post-carpal tunnel surgery or resolution of symptoms.

^a^ History of paraesthesia in the distribution of the median nerve, pain, swelling, weakness or clumsiness of the hand provoked or worsened by sleep, sustained hand or arm position, repetitive action of the hand or wrist that is mitigated by changing posture or by shaking the hand, and/or sensory deficits in the median innervated region of the hand, and/or motor deficit or atrophy of the median innervated thenar muscles; other causes excluded [3]

One researcher (MB), a clinician of 20 years of European descent with qualitative research training, conducted 40-60-minute interviews in person in local service or health settings, in the participant’s home or workplace, or in two cases by telephone. For three interviews, one or more family members were present. The interviewing researcher has strong connections and experience of the cultures of Māori and Pacific peoples in New Zealand and those with low and insecure income, through family, social, and occupational networks, including marae and the trade union movement. Her knowledge of the health system helped participants to make sense of the clinician workgroup outputs and recommendations. The researcher kept a diary and regularly talked with co-researchers to maintain a balanced viewpoint.

Interviews were audio-recorded and transcribed by the interviewing researcher in three cases and by a contracted transcription service (Pacific Transcription, Auckland NZ) for the remaining interviews. Field notes were made during and after the interview. Transcribed data were entered in NVivo data management software (NVivo 12– QSR International Pty Ltd, Melbourne) and subjected to deductive then inductive analysis by MB, using a systematic method of thematic analysis (Fig. 2) [40]. At intervals, coding, categorisation, and interpretation were discussed and reflected on with co-researchers. Participants were sent a 2-page summary of study findings and a 3-page detailed description of the themes and supporting quotes (step 8. of thematic analysis in Fig. 2). Permission was sought for the use of individual quotes, and feedback and comments invited on the themes and summary. Permission was declined for one quote regarding social position being an advantage in being offered an appointment. No further changes were suggested. Three responses commented on the strength of the findings and the value of patients being heard in the design process. A sub-group of four participants were subsequently involved in a series of consultation activities for how findings from the present study would be integrated in the HealthPathway development (reported in future work– Fig. 2).

Demographic and disease characteristics were collected using a tailored questionnaire. Participants completed self-report measures for symptom status and function (Boston Carpal Tunnel Questionnaire– BCTQ [41]). Although the BCTQ has been validated for a wide range of ages and nationalities, including Chinese, Korean, and Greek [42], the ethnicity(s) of the North American population in which it was developed [41] and that of a UK study [43] were undefined. The questionnaire therefore may have limited validity in the present study population. BCTQ scores were categorised by severity [44].

Participants were reimbursed for expenses incurred in participating in the study with a $25 petrol or grocery voucher. All names are pseudonyms.

## Results

Data were collected May to July 2021. Participant demographic and health characteristics are reported in Table 2.

**Table 2:** Participant demographic and health characteristics

Participant characteristics	*N* = 19 (%)^a^
Female	13 (68)
Age (years)	54 (SD 14.2)
Ethnicity	
New Zealand Māori	5 (26)
Pacific (Tongan)	1 (5)
Filipino	1 (5)
Bangladeshi	1 (5)
New Zealand European	11 (58)
Work status	
Paid	13 (68)
Unpaid	2 (11)
Retired	4 (21)
Income	
Low or insecure	10 (53)
Not low or insecure	6 (32)
Unsure	3 (16)
Rural domicile	9 (47)
Mean duration of symptoms (years)	5 (SD 6.0)
Care characteristics	
Carpal tunnel surgery in past 12 months	6 (32)
No treatment beyond referral with or without medication prescription	7 (37)
Other care^b^	6 (32)
Care funder	
Public	9 (53)
Private (self-funded) and Public	2 (11)
Private (self-funded and/or health insurance)	2 (11)
ACC (including accredited employer)	4 (21)
ACC initially, later declined and transferred to Public	2 (11)
BCTQ	
Symptom Severity Scale (11–55)	
Asymptomatic (11)	3 (16)
Mild (12–22)	2 (11)
Moderate (23–33)	5 (26)
Severe (34–44)	7 (37)
Very severe (45–55)	2 (11)
Functional Severity Scale (8–40)	
Asymptomatic (8)	4 (21)
Mild (9–16)	3 (16)
Moderate (17–24)	8 (42)
Severe (25–32)	2 (11)
Very severe (33–40)	2 (11)

ACC, Accident Compensation Corporation; BCTQ, Boston Carpal Tunnel Questionnaire

^a^ Percentages may not sum to 100 due to rounding

^b^ Other care = one or more of education and advice, exercises, physiotherapy, hand therapy, chiropractor, splint, corticosteroid injection, acupuncture

### Thematic analysis

**Fig. 3:**
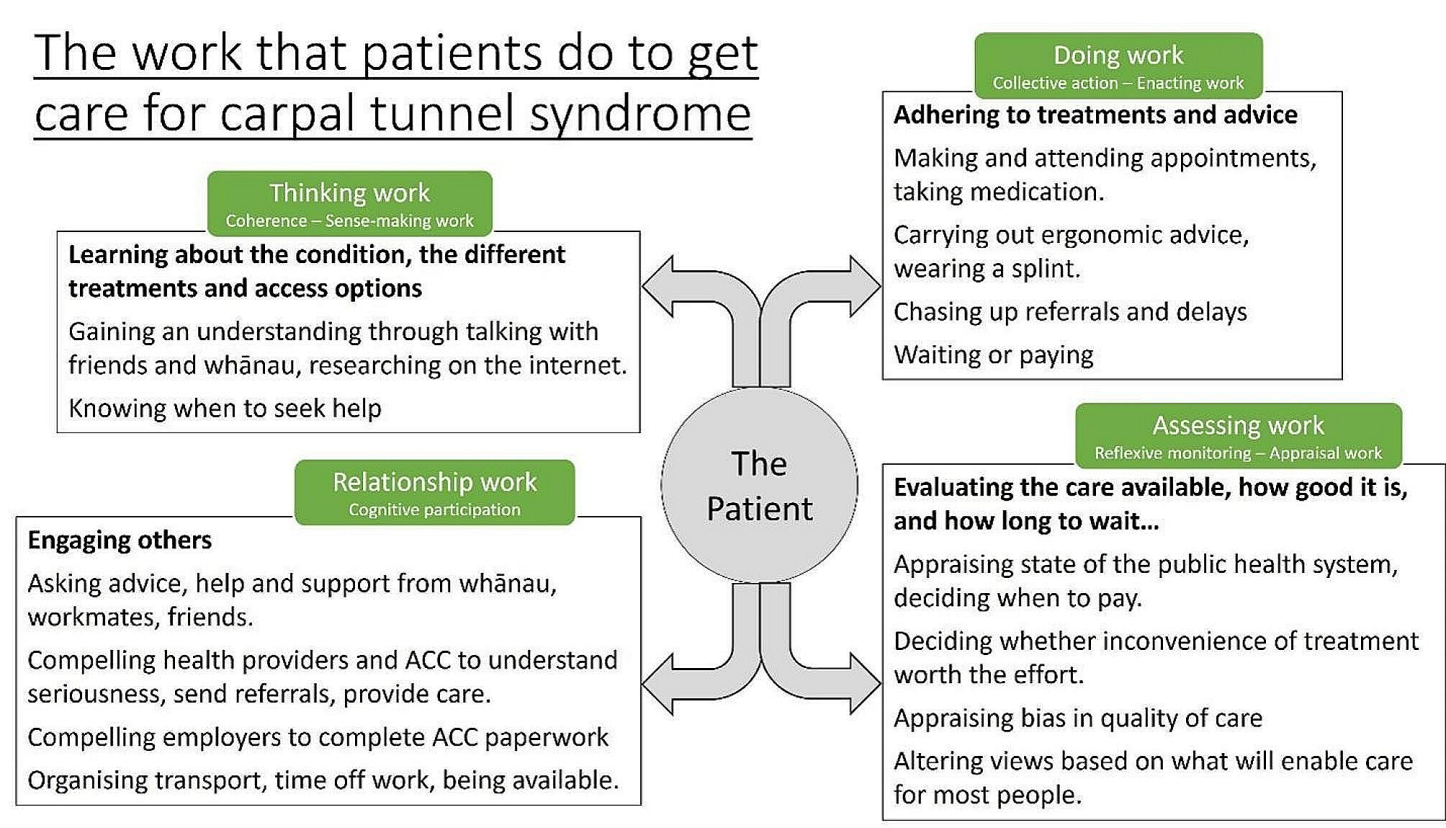
The work that patients do: overview of NPT analysis

Five major themes were identified: Significant Impact of CTS, Waiting and Paying for Care, Occupation and CTS Onset, CTS Information Scarcity, and Negotiating Telehealth Perspectives. Interpretation using NPT identified participant experiences relating to each of the four domains (Fig. 3).

1) The theme of ‘Significant Impact of CTS’ was characterised by the severity of symptoms, waking at night, worry about what will happen, yet finding it hard to be taken seriously. Participants found CTS to be a condition for which it was hard to make sense of the symptoms– if these would be temporary or lead to permanent harm, and how bad these might get. It was therefore often not evident that care should be sought early, yet also a cause for alarm about the future in terms of livelihood and the degree of impairment. *Charlene* was a 54-year-old shop owner who had experienced CTS for over 30 years since being pregnant with her first son. Although she had seen her GP many times, as well as other health providers, she remained uncertain about what was going on in her hands. Over the past year, symptoms had worsened: her hands were numb all of the time and pain kept her awake half the night, yet referral for care required multiple GP visits. There were financial and social impacts of having to employ someone to do part of her job and enlisting help from whānau [family], but it was her declining physical state and symptom severity that concerned her most.

*“What's going to happen, are they just going to shrivel up and fall off?” *

Viewed using the NPT domains, two types of work: sense-making (Coherence)– trying to understand the condition and deciding when to get care, as well as relational (Cognitive Participation)– compelling clinicians to provide care and to garner help from others, were seen to be high.

2) ‘Waiting and Paying for care’ refers to the delays to care associated with under resourcing in the public system, costs, poor quality of care, employment precarity, and burdensome ACC processes, in contrast to quicker, easier access when paying privately or through private health insurance.

*“[6 months ago]… he [GP] said well “you’ll never get an appointment at the hospital”. I don’t have um life insurance or medical insurance… you’re solely reliant on the public system…, like well I can’t sleep at night. I can’t lift, you know my hands are, one of them’s absolutely shot. Give us a hand here!”* Female < 65y.

Knowledge of how long the wait might be was important for reducing the added burden of trying to manage life in anticipation of care, and for making decisions about whether to pay– although for several participants private care was out of reach.

Poor quality care including racism, was another cause for delays and extra work by participants. *Tui-Helen*, a Māori participant living in a rural setting, described the extra effort she put in to avoid delays which she predicted might occur based on past experiences of seeking care in which racism was implicit.

*“…at the end of the day I’m not having them throw the excuse at me “you never came” because that seems to be the kaupapa [theme] that “…nobody turns up to the appointments”, or “you make an appointment they don’t come”, and I thought well no I made my appointment, I’m going! You know,…e mana-aki [show respect, generosity], just you know be humble, and a lot a karakia [prayer].”* Female, < 65yr.

*Tui-Helen* compensated for the unjust presumption made on the part of a health provider that she would be non-adherent or irresponsible, by making extra appointments, attending early, essentially engaging in what was being required of her by the health care providers to legitimate and speed up the route to access care.

*Gloria*, a 39-year-old manual worker from a rural setting, also experienced poor provider responses. She had symptoms of 3 years that were now interfering with her work and sleep.

*“I said to my doctor, over the years on several occasions, "Oh I've got this problem… I’ve got this problem" and she's keep fobbing me off "Oh we'll keep an eye on it, keep an eye on it".” *

These experiences reflect the NPT domains of Cognitive Participation (Enrolment, Activation, and Legitimation)and Collective Action (Relational integration and Contextual integration– the resourcing of time and consultation fees) to compel clinicians and health services to provide care, as well as Reflexive Monitoring to appraise the quality of care.

For Trina, a 38-year-old mum and part-time labourer, it was precarious employment that led her to decline referral for investigation and delay her care.

*“If I actually knew for myself that it is or it isn’t carpal tunnel and then if it is, I mean, job prospects aside, I’d better deal with it before it gets sore again. I know what I’m doing [declining investigation and diagnosis] is not the right choice but life is busy and things are hard.”*

Difficult ACC processes were another reason for long delays. The onus of responsibility on the participant to get the ‘proof’ [that symptom onset was work-related] was particularly fraught for those in small or unsupportive employment environments.

*“I’ve got a form from a doctor to go through ACC and he’s got [date 18 months ago]… I have to send them on to… whoever is I’ve been working for to fill out. And then the person at ACC said to me “oh we’ll probably reject them"… he [GP] said “oh if they reject them we’ll just apply again“… in all honesty I need to take these forms up to get them sorted… I just been so busy,… the [employer] and I don’t get on…”* Female < 65yr.

In contrast, a supportive work environment and strong relationships with and between health providers moderated participants’ work to compel ACC or other health services to provide care. *Joseph*, a 63-year-old who works in a meat processing plant with an active union presence and health providers onsite described his positive experience accessing care:

*“It was that letter [health provider] wrote, it was clear evidence the length that I went because the carpal tunnel... the beauty of this, [health provider] knows the surgeon. [Health provider] knows the doctor and he write them… the help they need [to give me].” *

3) ‘Occupation and CTS Onset’ identified symptoms that were associated with current or previous work in a wide range of industries, yet little acceptance of this by ACC. *Melanie*, a 25-year-old Māori participant from a rural town, described her onset of symptoms as the intensity of her work in cleaning and kitchen handing increased.

Social position could also bring advantage, for example *Celia*, a 54-year-old health professional in a rural town noted that she was lucky because her position as a health sector worker probably contributed to her being offered an appointment (in the private sector) reasonably quickly.

*“It was from working. I was working one job, doing cleaning and then I started another secondary job, washing dishes and that's when it started, when I was doing both jobs.” *

That ACC processes were “too difficult” was a common reason given by participants for either them or their health provider deciding not to lodge a claim. Participants also shared stories of family and friends who had ‘fought’ with ACC and the enormous effort involved, which informed participants’ own experiences and choices.

*“[My brother] had surgery when he was about 21. Yes, he had to wait quite a long time to get that surgery. He had to fight a lot for it, because no one would take responsibility for it. So, they were trying to get it under ACC, because of been working in the McDonalds. That didn’t work out. So, he had to go through public… he had to wait on a very, very long waiting list.”* Female, < 65yr.

*“I have heard of another case, a friend, she was a fish filleter and she got it and then her claim was turned down, but she had to fight for it.”* Male, < 65yr.

Claims were declined because of insufficient evidence that the occupation has caused the problem… to prove that occupation directly contributed to the problem is incredibly difficult with something of a gradual onset nature. The substantial relational work– Initiation, Enrolment, Legitimation, in addition to Enacting work involved in engaging with ACC was beyond the capacity of most participants.

4) ‘CTS Information Scarcity’ describes how good information about CTS is hard to find and rarely offered unless in the context of an extended private specialist appointment.

Julie-Anne, a 53-year-old mum and small farmlet operator, reflected that over her 10-year history of CTS including one surgery 7 years ago and a second more recently, no one had asked her about the chain sawing and heavy impact work she regularly did, or discussed how this might relate to her condition.

*“I think yeah, if I had done that [activity modification and splint] earlier on... At that point, you could have information about what causes it and the things you can do to help yourself.” *

Participants were unanimous in their desire for information about CT and its management. However, some who searched online found that the information often lacked relevance.

*“A lot of the times the American websites come up and you think, I put New Zealand in, and still American sites come up. You’re this and… I don’t know, I don’t know.”* Female, < 65yr.

Going online introduced additional relational and sense-making work– avoiding unsolicited notifications or advertising and judging what was accurate versus inaccurate information. On the other hand, social media was emphasised to be a highly valued source of health information by the participant from a Pacific community, because of language accessibility, communicative style, and trust in the community.

5) ‘Negotiating Telehealth Perspectives’ captured a range of views in which the role of telehealth in a future pathway was generally viewed positively and capable of serving the communication purpose, although it was highlighted that this may not work for some such as older relatives or kaumātua (elders) or those with connection difficulties.

*“That would work! That would absolutely work… But the kaumātua need to be seen. Kanohi-ki-te-kanohi [face-to-face], and not put on the devices”* Female < 65yr.

On the other hand, it was seen as a complete *“cop-out”* by a few. Several rural participants preferred to drive several hundred kilometres rather than use telehealth for a specialist assessment, although this also reflected their desperation to get something done.

In-person appointment was preferred by most, in part due to concerns about a virtual consultation being inadequate in the context of a physical problem and a sense that personal validation and important communication may be lost.

*“So, if you do that one, if the surgeon will do that one through video, I can fake it or I can how can he touch my… hands?”* Male < 65yr.

*“A lot of decisions are made on body language and how you fold your arms or shut yourself off or something. So, that’s the things to take into account when you’re talking not face-to-face visits.”* Male < 65yr.

Yet all except one participant would use telehealth if it reduced the wait and improved access for all. Financial assistance was pointed out as necessary for telehealth to be accessible– this was available from Work and Income New Zealand (social welfare) for a television but not yet for laptop computers.

The Cognitive Participation (relational work), Collective Action, specifically Interactional workability and Relational integration, and Contextual integration (resourcing) of telehealth requires individual consideration.

A sixth, minor theme, ‘Complexity in Simplicity’ described the incongruency between the simplicity of the surgical procedure (CT release) and the enormous difficulty of accessing the care.

*“How bad is this going to get before anyone’s gonna, just snip! You know, like.”* Female, < 65yr.

## Discussion

Major themes identified in this study were the wider impact and onset of CTS, delays and extensive waiting for care in the public system ameliorated by paying for private care, lack of information about CTS, and weighing up views on telehealth. NPT interpretation identified that in accessing and engaging care for CTS, those with greater privilege – economic, relational (social capital), or secure and supportive employment conditions, are required to do less work while those with less privilege have substantially more work to do. The latter group often work exceptionally hard and nevertheless may still not get care. These findings add to an existing body of literature that demonstrates how social and economic inequality contributes to inequities in health care access and outcomes, particularly where health services fail to effectively respond to peoples’ circumstances [45–48].

This study suggests that even for a relatively simple condition such as CTS, addressing economic as well as provider and system-related factors such as language and communicative styles, values, and beliefs including ethnic bias, is necessary to help eliminate inequity. Moreover, a broad view across the health system, including private care and ACC, is necessary to adequately delineate equity issues. Increasing access to publicly funded CT surgery would reduce the major impact of waiting, particularly for the groups prioritised in this study. Workplaces are key arenas for change– mandated health services in or adjacent to workplaces, leave from work for health appointments, and changes to ACC processes for fairer, quicker access to care. Robust preventive and health promotion programmes could reduce the impact of CT and consequently the need for care.

The potential for telehealth to reduce patient burden in terms of transportation, time, and work interference, as well as the negative impacts of racism by shifting the power imbalance away from clinicians, were valued by participants. However, concerns about remote physical examination, absence of doctors’ hands, and lack of rapport, as previously found [49, 50] meant some patients felt they would be more easily dismissed as the extent of their symptoms may not be so evident. A structured, culturally responsive communication approach such as the ‘Hui Process’ [51, 52] and explanation of the evidence for telehealth including any known limitations, may address these concerns. Access to technical support, devices, and connectivity is also necessary for equitable access and experience [50, 52].

Delays in seeking care associated with perceived low seriousness of CTS is a finding of both the present and previous studies [53, 54]. This may be partly explained by the high sense making work, i.e., the difficulty of understanding the impact of occupation and positioning on nerve physiology and the potential long-term consequences. Quality information, clear processes for when and how to seek further care, and clinician time to tailor information to individuals, would reduce this work for patients and improve care access and health outcomes. Clinical decision-making and prioritisation must also account for the incongruency to avoid wrongly denying care.

### Strengths and weaknesses of the theory

**Fig. 4:**
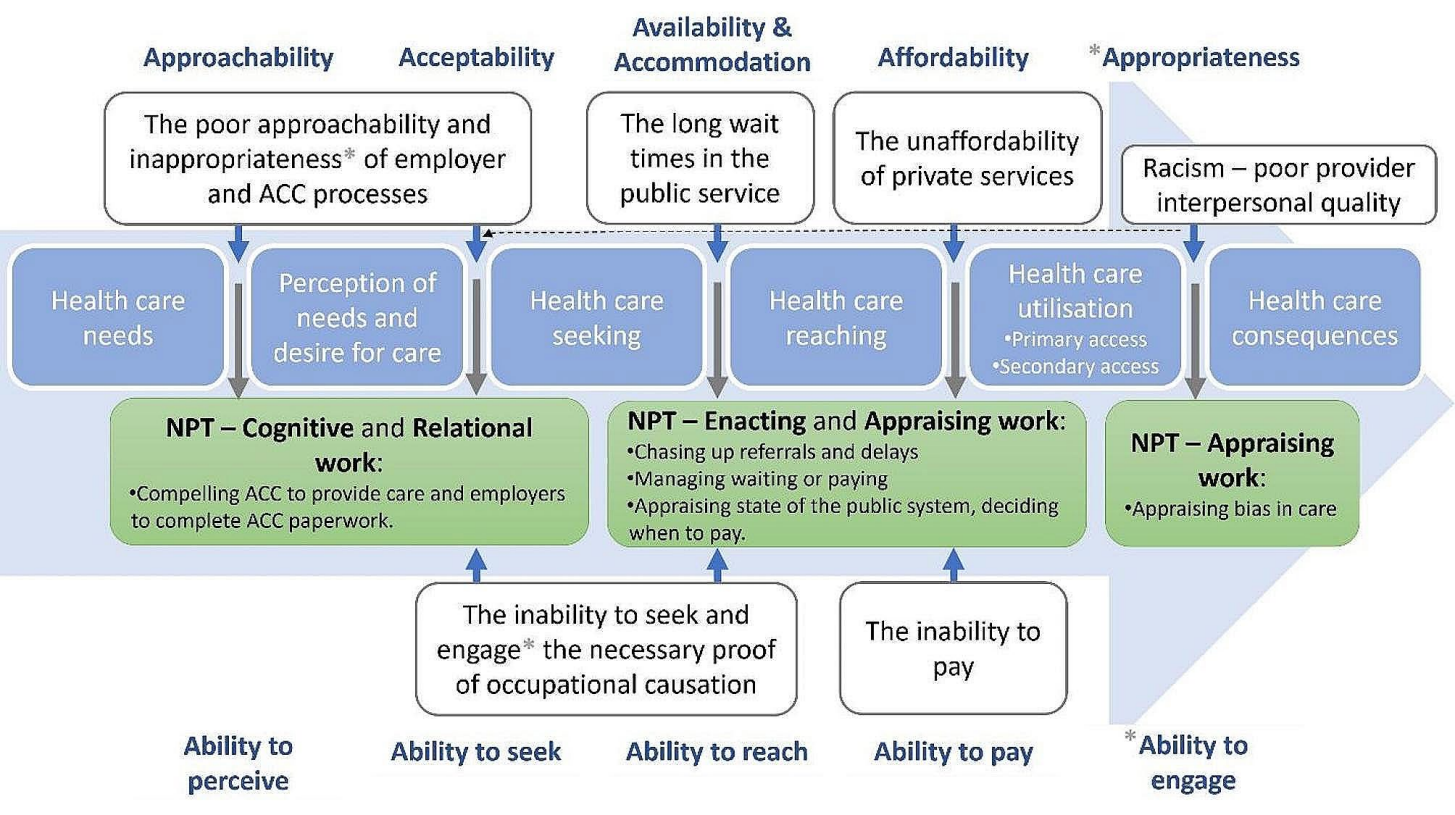
Examples of patient experiences and resulting NPT analysis of patient work (black text) aligned to the Levesque et al. [55] framework of health care access (blue and white text)

As a theoretical framework for interrogating the labour involved in seeking health care, NPT usefully identified components of patient ‘work’ as distinct from illness burden and where the differences between the groups relevant to the inequity e.g., lower rates of accessing ACC services for Māori compared to non-Māori [10] are apparent, helping to explain how inequity operates. The framework helped to make sense of the findings by considering the interrelationship between social and structural constraints and individual agency. In this respect, it aligns with the conceptual framework of health care access developed by Levesque, Harris & Russell (2013) [55] which seeks to understand the relationships that occur at the interface of health systems and populations. Our NPT analysis prioritises the patient perspective of these relationships and reveals the resulting burden lumbered on patients (Fig. 4).

NPT thus gives insight into the dynamics of the health system-population interface, including the impact of its nature, be that bureaucratic, digital, or human, and the influence of other players (such as unions and employers).

The NPT-derived understanding of work that results from experiencing racism is particularly valuable as it recognises the enormous efforts made in seeking care and highlights the need for health services to start meeting patients halfway. Racism is a major driver of unequal access to care across countries, health care indicators, and health care settings [56, 57]. Our NPT analysis would suggest that racism operates within the Levesque concept of ‘appropriateness’ of care, as a failure of provider interpersonal quality with flow on effects back to ‘availability’ and ‘acceptability’ (Fig. 4).

The findings of this study confirm the importance of interventions proposed by previous studies to support earlier access to care and achieving health equity. These include: additional clinician time and resources [58, 59]; culturally safe and responsive communicative skills and tools [58, 60, 61]; patient support with costs and fees [47, 59, 61, 62], devices and connectivity [50, 59, 63]; tailoring support to individuals [61]; and preventive programmes [58, 62]. Embedding cultural safety training that enables clinicians to acknowledge the inherent power differentials between provider and patient is recommended for addressing clinician bias, particularly ethnic bias [56, 64], although training alone is unlikely to address racism entirely due to its structural nature [56].

The need to go beyond interpersonal relations and change systems, policies, and resourcing has also been emphasised by numerous authors [47, 62]. Strengthening employment relations is a more novel recommendation from this study and may have a more wide-reaching impact on health equity, particularly for those with low or insecure income [18]. The more nuanced understanding of the relative merits of telehealth is another strength.

NPT-informed participatory interview methods are a practical aid for assessing service equity and access and evaluating who is advantaged and how from the patient perspective. This approach warrants further investigation, perhaps integrated within other tools, such as the New Zealand Health Equity Assessment Tool [62] and the New Zealand Co-design Toolkit [33].

### Strengths and limitations overall

This study was unusual for NPT in that it focussed primarily on the perspectives of patients. While not multi-perspectival in its data gathering, it reinforces the message that the implementation of health interventions in complex health systems owes as much to the work of patients as it does to service providers and other personnel [65]. The inductive, in addition to deductive analysis allowed for a range of views and issues to be identified, and helped overcome challenges previously associated with the theory around fitting codes and categories solely on a predetermined conceptual framework [66].

A strength of this study is the focus on populations more likely to underuse health services, including those with low or insecure income, unusual in studies of musculoskeletal care. While recruitment met other predefined demographic and care characteristic targets, only one Pacific participant was recruited. A focus on Pacific peoples is a priority for a future study. This will include Pacific language speakers among the interview team or available as a translation service. Partnering more closely with Pacific community groups will support greater Pacific involvement in the research and service design.

While we have found NPT to be useful in informing health service (and system and policy) design that may potentially contribute to eliminating inequities, whether these elements in fact deliver this, and how they can be implemented and evaluated, are future questions.

## Conclusion

Experiences and perspectives of health services for CTS among those more likely to underuse services suggest poorer quality and less timely treatment for those unable to pay for expedited care and extended consultations. Our study findings and NPT analysis suggest a range of interventions that are needed to enable greater health service mitigation of the ongoing impact of wider social and economic inequalities. Racism, conceptualised as a problem of quality of care, is a critical target for reducing inequities in health care access; more work is required to understand and address racism from a structural perspective.

Our use of NPT in patient co-design interviews has been helpful for defining the specific problems to be overcome to achieve equitable early access to care. We recommend NPT for prospective use in future service development.

### Electronic supplementary material

Below is the link to the electronic supplementary material.

Supplementary Material 1

## Data Availability

The datasets used and/or analysed during the current study available from the corresponding author on reasonable request.
