# Supplementary material for: Patient experiences and perspectives of health service access for carpal tunnel syndrome in Aotearoa New Zealand: a normalisation process theory-informed qualitative study
Source: BMC Health Serv Res. 2024 Apr 13;24:465. doi: 10.1186/s12913-024-10871-x (PMC11015558; doi:10.1186/s12913-024-10871-x)
Supplement: Supplementary file 1 — Supplementary Material 1 [file 12913_2024_10871_MOESM1_ESM.pdf]

Supplemental 1 Interview schedule

1. Introduction
    a. Welcome the participant(s) and introduce the interviewer (XX).
    b. Consider opening and closing with a karakia *[prayer]*
    c. Explain about the research and the purpose of the interview. Opportunity for participants to introduce themselves.
    d. Manaakitanga *[showing respect, generosity – for example, through hospitality]*: cup of tea, light refreshment
    e. Outline the format of the interview including use of a visual tool (Whakāro Pōkare – a rippling pool)[1] to invite participants to reflect on the impact of their carpal tunnl problem with respect to the four pillars of health – tinana [physical], wairua [spiritual], hinengaro [thoughts and feelings], and whānau [family/community] as represented in the New Zealand Māori holistic health framework of Te Whare Tapa Whā,[2] familiar to most New Zealanders.
    f. Opportunity for any questions
2. Procedural information
    a. No right or wrong answers
    b. You can change your mind
    c. Can take 'time-out' at any time, without needing to give any reason
    d. The interview will be recorded, are you ok with that?
    e. Any information that could identify you will not be disclosed to anyone other than the researcher (XX), so please speak freely and fully.
    f. Opportunity for any further questions
    g. Whakawhanaungatanga [relationship building]: ice breaker – *favourite place to go on holiday; best thing about Southern district.* Allow some time for discussion to get to know one another and the facilitator.
    h. Confirm consent to participate; indicate recording will start.

3. The interview questions

**Patient story**
Can you tell me about your carpal tunnel problem?<br>Can you tell me more about that?<br>How long ago did that start?<br>Have you used any non-prescription (e.g., bought at the supermarket) pain killers, Rongoa [natural remedy], or other pain relief, e.g., alcohol or marijuana for your carpal tunnel problem?<br>What changes have you had to make… at home, work, usual activities?<br>What impact has that had? (Whakāro Pōkare tool – whānau, tinana, hinengaro, wairua)
**Journey map and Contact Points**
Can you tell me about what care or information you have sought or received?<br>Can you tell me more about that?<br>Who did you see about your hand (GP, nurse, physio, hand therapist, specialist, internet/Google) [*substitute for 'provider' for interview*], when?<br>What type of visit was it? (In-person visit, Video call, Phone call, Email, Text message)<br>What was main purpose of the visit? (Getting advice or information, Assessment, Getting a prescription?)<br>If more than one appointment, what was most recent? (In-person visit, Phone call, Video call); most frequent?

**Patient Stories and Hotspots – *experience***
Can you tell me about your personal experience of your visit with the [health provider]<br>Do you get to see your usual [provider]? (e.g., same GP?)<br>When you made the booking, how quickly were you able to get an appointment?<br>How did you feel about the wait?<br>Do you find that the reception and/or admin staff treated you with respect?<br>Did you need an interpreter to communicate with the health provider? How did you find that?<br>Did your [provider] listen to you? Inform you as much as you wanted about your health condition and your care including medications? Explain things in a way you could understand? Treat you with kindness and understanding? Spend enough time with you? Spend too much time with you?<br>Did the healthcare provider involve you as much as you wanted to be in making decisions about your treatment and care?<br>How would you rate this [provider]'s knowledge of your values and beliefs that are important to your health care?<br>Did you feel your individual and/or cultural needs were met?<br>How could your individual and/or cultural needs have been better met?
**Patient Stories and Hotspots – *access***
Can you tell me about your experience accessing health services and information<br>How much did your visits cost? Did you have to take time off work / jobs at home? Did you need help from others?<br>Have you heard of or used a general practice online service or patient portal (e.g., ManageMyHealth, Health365, ConnectMed, OpenNotes)? *These can be used to book appointments, order repeat prescriptions or to see your health records.*<br>Have you had a look at your portal to view information about your hand or to contact your [health provider]?<br>Was there ever a time when you wanted to see someone about your hand but couldn't? (Y/N) If Yes:<br>Why could you not get health care from a provider when you wanted it? *(e.g., Waiting time to get an appointment too long; The appointment was too expensive; Owed money to the general practice or medical centre; Dislike or fear the provider; Difficult to take time off work; Had no transport to get there; Could not arrange childcare or care for a dependent; Did not have support person or interpreter to go with; Unable to use the technology; No access to the technology; Fear of getting sick by visiting in person; Alert level restrictions meant I wasn't allowed; I didn't want to make the health care providers too busy; I was worried about catching COVID-19; Other...)*<br>If you have seen, or have been referred to see a specialist, were you seen as soon as you needed to be seen?<br>Was the cost of seeing any of the providers difficult for you to afford?
**Patient Stories and Hotspots – *care integration and engagement***
Can you tell me about quality of communication you experienced?<br>What went well… what could have been better?<br>Thinking back about the care you got, how often do you think your [provider] understood the things that really matter to you about your hand? About your health generally? About your non-prescription pain killers?<br>How often did your [provider] ask you for your ideas about managing your hand?<br>Did this [provider] or someone in their office ask you about things that make it hard for you to take care of your hand, and did they help you to come up with a plan to help you deal with these things that make it hard for you to take care of your health?<br>Did they give you instructions about how to take care of your hand problem, and did the instructions help you take care of your hand?<br>Did your [provider] talk to you about what is available in your neighbourhood or online to support you in managing your hand?

Did you ever leave your [provider's] office confused about what to do next to manage your carpal tunnel problem?<br>If you had a question about managing your hand today, would you know how to get answers you trust?<br>When this [provider] or someone in their office ordered a blood test, x-ray, nerve conduction study, or other test for you, did they follow up to give you those results?<br>Was it easy to understand your test results?<br>Did this [provider] talk with you about how you were supposed to take any medicine prescribed or follow advice given for your hand?<br>Have you found or received any information about how to manage your carpal tunnel problem?<br>Where did you find it or who gave it to you?<br>Was it easy to understand?<br>Can you remember what was the most useful part of it?<br>Did you ever find or receive information about managing your hand that disagreed with what another provider or information told you to do? How was that?<br>If you have seen a specialist, how often do you have to repeat information that you have already given to the [first provider] you saw?<br>When you see this specialist, do they seem to know enough information about your carpal tunnel and your medical history?<br>In general, do you think the [providers] that you see/saw for your hand communicated with each other about your care?<br>If you have had surgery or another procedure e.g., injection for your carpal tunnel, did you know what to expect after and did the instructions you were given make you confident about caring for your hand?<br>As far as you know, was your GP / nurse clinic informed and up to date about the plan for follow-up?<br>Did you have to go back to hospital or get emergency care because of complications or your condition got worse within a month after the procedure? What was that like?
**Perspectives on future pathway – clinician work group output**
A clinician work group from Southern DHB and primary care have developed these ideas about a care pathway for carpal tunnel syndrome [visual output].<br>*Spend 2-3 minutes describing the key points in the clinician work group output.*<br>*Highlight patient contact points, types of contact, and with whom. Provide visual information as printout or onscreen.*<br>Are there any ideas you think might not work well? What might work better?
**Perspectives on future pathway – potential roles for telehealth**
Telephone consultation can involve a discussion with a health care provider with opportunities to ask questions and receive information and referral if necessary.<br>*A health provider or you can decide if you need to be seen in-person as well.*<br>Do you think a telephone appointment would work for you?<br>What do you think would make it a good experience for you?<br>Where do you think it would work? (e.g., GP; hotline; physiotherapy; getting a call from the hospital for more information, or an assessment for surgery follow-up care)<br>*Video consultation is similar to telephone but you can see one another. It needs access to a smart phone or computer with a camera, microphone, and speaker or headphones, and a link to the secure online video platform. You usually need to share your email address with the health provider.*<br>Do you have access to this equipment?<br>How confident would you be to use this equipment?<br>How do you feel about sharing your email address?<br>Do you think a video appointment would work for you?<br>What do you think would make it a good experience for you?

Where do you think it might work for you? (E.g., GP; hotline; physiotherapy; getting a call from the hospital for more information, or an assessment for surgery; follow-up care)
**Perspectives on future pathway – web-based information**
Do you think getting information online about carpal tunnel would work for you?<br>How would you feel about getting information about your carpal tunnel problem online? (e.g. NZ Health Navigator, other patient information website, online health service)<br>What do you think this should look like (e.g., pictures or videos versus writing? What would make it easier to find?<br>What would be the way you would like to receive information about carpal tunnel syndrome?
**Perspectives on future pathway – quality of care**
Are there any other changes or ideas that you think would make the care better?<br>I.e., that would make it a better experience? That would have made your hand better quicker? Make it easier to get care?
**Perspectives on pathway constructs (NPT)**
In thinking about a potential future pathway of care for carpal tunnel syndrome,<br>a. What would you describe as a good experience of care? A good health outcome? Care that is easy to get?<br>b. What things do you think need to be part of a pathway to make it easy to get care and get a good outcome?<br>c. Do you think what the clinician work group has suggested will help these things to happen?<br>Which bits do you think would help you to communicate well with your [provider], *other providers*?<br>Which bits do you think would help you trust your [provider]?<br>Can you think of any other things necessary for the care pathway to work well?
**Perspectives on pathway evaluation methods**
Collecting feedback and outcome measures helps to continue improving health services.<br>What do you think about completing questionnaires or surveys about your health outcome and care experience?<br>Do you think it would work for you to give feedback about your experience of care on a computer or smartphone? Complete an outcome measure?<br>Would you prefer paper or electronic?<br>Would you prefer an electronic link to complete at home online or via access through your patient portal? Or on a device at a health centre?
**Most important change**
What is the most important thing you would change 1) about the impact carpal tunnel symptoms have had for you, and 2) about your experience of care?<br>Is there anything else you would like to add?<br>Reflect on impact using the Whakāro Pōkare tool<br>Reflect on experience of care
**INTERVIEW ENDED**

- Complete demographic and disease information (x)
- Draw the session to a close and review what happens next (per Participant Information Sheet).

References

1. Bryant KAP. *He kiteka hauā i Murihiki*. University of Otago; 2016.
2. Durie M. *Whaiora: Māori health development*. 2nd ed. Oxford University Press; 1998.
